# Effectiveness of a low-dose gonadotropin protocol for controlled ovarian stimulation at a University Assisted Reproduction Center

**DOI:** 10.1016/j.clinsp.2026.101178

**Published:** 2026-10-06

**Authors:** Mayra Satiko Lemos Nakano, Edmund Chada Baracat, José Maria Soares Júnior, Henrique Penna Fazão, Tatiana Carvalho de Souza Bonetti, Pedro Augusto Araújo Monteleone

**Affiliations:** a Centro de Reprodução Humana Governador Mario Covas, Disciplina de Ginecologia, Departamento de Obstetrícia e Ginecologia, Hospital das Clínicas, Faculdade de Medicina da Universidade de São Paulo (HCFMUSP), São Paulo, SP, Brazil; b Centro de Reprodução Humana Monteleone, São Paulo, SP, Brazil; c Departamento de Ginecologia, Escola Paulista de Medicina, Universidade Federal de São Paulo (EPM-UNIFESP), São Paulo, SP, Brazil

**Keywords:** Controlled ovarian stimulation, Low-dose protocol, IVF, Clinical pregnancy, Gonadotropins

## Abstract

**Objective:**

This study evaluates the effectiveness of a low-dose gonadotropin protocol for Controlled Ovarian Stimulation (COS) in *In Vitro* Fertilization (IVF) for patients with a good prognosis attending a public health university care center.

**Study design:**

A randomized, non-inferiority, single-blind clinical trial. This study included 90 couples undergoing IVF cycles. Patients were randomized to receive either the regular-dose (REG-DOSE, n = 46) or low-dose (LOW-DOSE, n = 44) gonadotropin protocol. The REG-DOSE group received menotropin (225 IU/day), while the LOW-DOSE group received the combination of clomiphene citrate (Clomid®) and menotropin (150 IU/day). The primary outcome was the number of blastocysts available for transfer.

**Results:**

The REG-DOSE and LOW-DOSE groups had similar blastocyst development rates (49.9% and 54.8%; p = 0.463). The secondary outcomes measured, such as ongoing pregnancy rates after the first embryo transfer (23.7% and 26.5%; p = 0.729) and the cumulative clinical pregnancy rates (34% and 30%; p = 0.726), respectively, were also comparable.

**Conclusions:**

Results provide strong evidence that low-dose gonadotropin protocols for COS in good prognosis IVF patients yield comparable clinical outcomes to regular-dose protocols, with the added benefit of reduced treatment costs. These results support the clinical equivalence of low-dose protocols in selected patients and highlight their potential to reduce costs and increase access to IVF, particularly in public healthcare systems.

## Highlights

• Low-dose gonadotropin stimulation achieved non-inferior IVF outcomes.
• Blastocyst development was maintained despite lower gonadotropin exposure.
• Pregnancy rates were comparable between low- and regular-dose protocols.
• Reduced gonadotropin use may lower IVF costs without compromising efficacy.
• Low-dose IVF could expand access to fertility treatment in public healthcare centers.

## Introduction

*In Vitro* Fertilization (IVF), first pioneered with the birth of Louise Brown in 1978, is now widely indicated for a variety of infertility causes, including female or male infertility, genetic disorders, and fertility preservation for social or medical reasons. A critical component of IVF is Controlled Ovarian Stimulation (COS), which aims to induce the development of multiple oocytes. While conventional protocols often use high-dose gonadotropins to maximize oocyte retrieval,^1^ evidence suggests that lower doses may yield similar clinical outcomes, with the added benefits of reduced costs and improved patient access to treatment. Recent studies, including the OPTMIST trial, demonstrate that lower doses of gonadotropins may be as effective as higher doses for patients who are hyper-responsive to stimulation, offering comparable live birth rates despite fewer oocytes being retrieved.^2^, ^3^, ^4^

Furthermore, the increased cost of regular gonadotropin protocols limits accessibility, particularly in public health systems. Therefore, when the focus is on improving access to assisted reproduction treatment, low-dose protocols are supposed to be an excellent option for patients with a good prognosis. This study aimed to assess the efficacy and cost-effectiveness of a low-dose gonadotropin protocol for COS in good prognosis IVF patients at a university-affiliated assisted reproduction center.

## Methods

### Study design

This randomized, non-inferiority, single-blinded clinical trial was conducted at the Human Reproduction Center Governador Mário Covas, Hospital das Clínicas, da Faculdade de Medicina da Universidade de São Paulo (HC-FMUSP), from June 2018 to June 2022. The study received ethical approval from the Research Ethics Committee (Protocol 2.783.961 and CAAE 9244621850000006) and was registered with ReBEC (Protocol RBR-2xxcvqj). Written informed consent was obtained from all participants.

This study included infertile couples undergoing IVF treatment who presented with a good prognosis, as following inclusion criteria: women aged 18‒38 years with a Body Mass Index (BMI) ≤ 30 kg/m^2^, basal FSH < 10 mIU/mL, and at least 5 antral follicles on day-3 of the menstrual cycle. Couples who required sperm collection by surgical methods were excluded. The primary outcomes included the number of blastocysts available for transfer. Secondary outcomes included cycle cancellation rate, clinical pregnancy rate per initiated cycle and per embryo transfer, miscarriage rate, multiple pregnancy rate, and Ovarian Hyperstimulation Syndrome (OHSS) incidence.

#### Sample

A sample size calculation was obtained by a non-inferiority test, based on the primary outcome of the number of blastocysts developed and available for transfer. As the patients in this study have a good prognosis, we estimated that the standard group (REG-DOSE) would have an average of four blastocysts available for transfer. Based on the concept that the maximum chance of a successful pregnancy in an IVF cycle is reached after three blastocysts transfers, we considered a difference of one blastocyst available as a non-inferiority limit for the study group (LOW-DOSE). Suppose there is truly no difference between the standard (REG-DOSE) and experimental treatment (LOW-DOSE), then 50 patients per group (100 patients) are required to be 80% sure that the lower limit of a one-sided 95% Confidence Interval (or equivalently a 90% two-sided confidence interval) will be above the non-inferiority limit of -1. The non-inferiority margin of one blastocyst was defined a priori based on clinical rather than statistical considerations. Considering that cumulative live birth rates increase substantially with the availability of multiple blastocysts but tend to plateau after approximately three embryo transfers, we considered that a reduction of one blastocyst would not be expected to result in a clinically meaningful decrease in the cumulative chance of live birth. Therefore, an absolute margin of one blastocyst was considered clinically acceptable while preserving the expected reproductive benefit. This approach is consistent with recommendations that non-inferiority margins should be based on clinical judgment supported by available evidence rather than solely on statistical criteria.Sample size calculation and randomization used the Sealed Envelope^TM^ software (https://www.sealedenvelope.com/). A total of 90 patients were randomized as 46 at REG-DOSE and 44 at the LOW-DOSE groups because this study was conducted during COVID-19 pandemic season and our Hospital was reference for COVID-19 patients, for that reason the Human Reproduction Center was closed for new cycles in 2020 and 2021.

### Interventions

All couples meeting the inclusion criteria who were starting IVF cycles during the study period were invited to participate and were randomly assigned before beginning the IVF treatment, in a 1:1 ratio, to either the REG-DOSE or LOW-DOSE groups. The REG-DOSE group received 225 IU of Menotropin (Menopur®) daily, while the LOW-DOSE group received 100 mg Clomiphene Citrate (Clomid®) for five days, followed by 150 IU Menotropin (Menopur®) from day three of stimulation. All procedures performed after COS were performed according to the routine of the IVF center in both groups. Briefly, GnRH antagonist (Cetrotide®) was administered for pituitary blockage and hCG (alpha choriogonadotropin, Ovidrel®) or GnRH agonist (Gonapeptyl®) were alternatively used for oocyte maturation trigger. The triggering criteria was the risk of ovarian hyperstimulation syndrome, as serum estradiol was > 3,500 pg/mL or > 20 follicles greater than 14 mm determined the GnRH agonist triggering; except for those situations, the trigger with hCG was used. Oocyte retrieval was performed 35‒36 hours post-trigger. Fig. 1 shows the ovarian stimulation protocol.

**Fig. 1:**
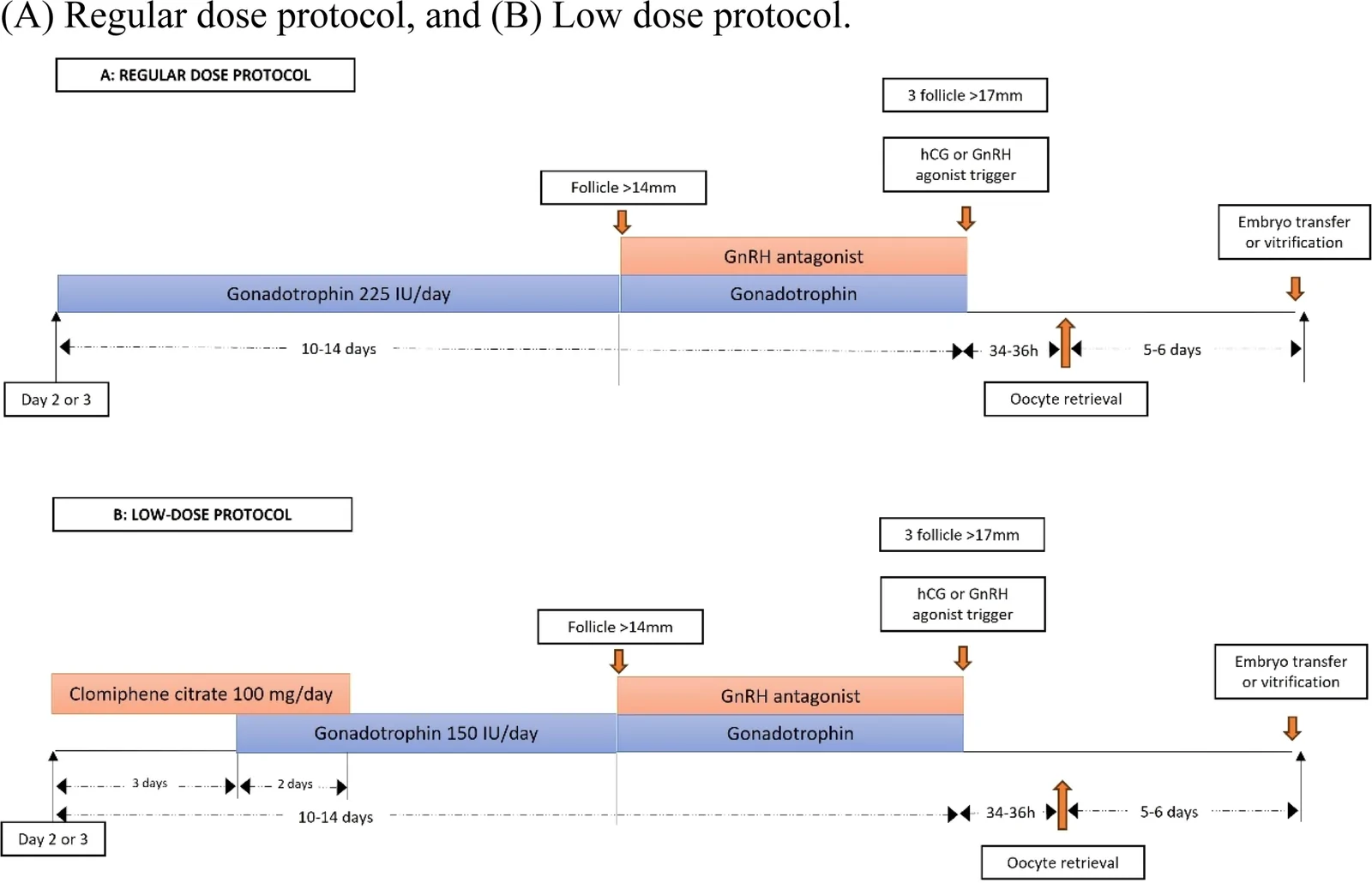
Diagram illustrating the controlled ovarian stimulation protocols for the groups. (A) Regular dose protocol, and (B) Low dose protocol.

Intracytoplasmic Sperm Injection (ICSI) was used for fertilization of all Mature oocytes (MII) and embryos were cultured to the blastocyst stage following routine protocols. For transfer, one or two embryos were transferred, based on clinical indications and patient preference. Surplus embryos were cryopreserved using the vitrification method (Vitrification Freeze kit, Irvine Scientific). For cases who presented any risk of ovarian hyperstimulation syndrome, all embryos were cryopreserved using the vitrification method (Vitrification Freeze kit, Irvine Scientific) at the blastocyst stage, and the embryo transfer was placed in a subsequent hormone replacement cycle.

For couples with no pregnancy after the first transfer, a second transfer could be performed. For Frozen-thawed Embryo Transfers (FET), the endometrium was stimulated with estradiol valerate (8 mg, Primogyna®, Bayer) until endometrial thickness > 7 mm. Vaginal micronized progesterone (800 mg, Utrogestan®, Besins) was used for five days before the FET. The embryology laboratory team was blinded to the study group. Fig. 2 shows the study design.

**Fig. 2:**
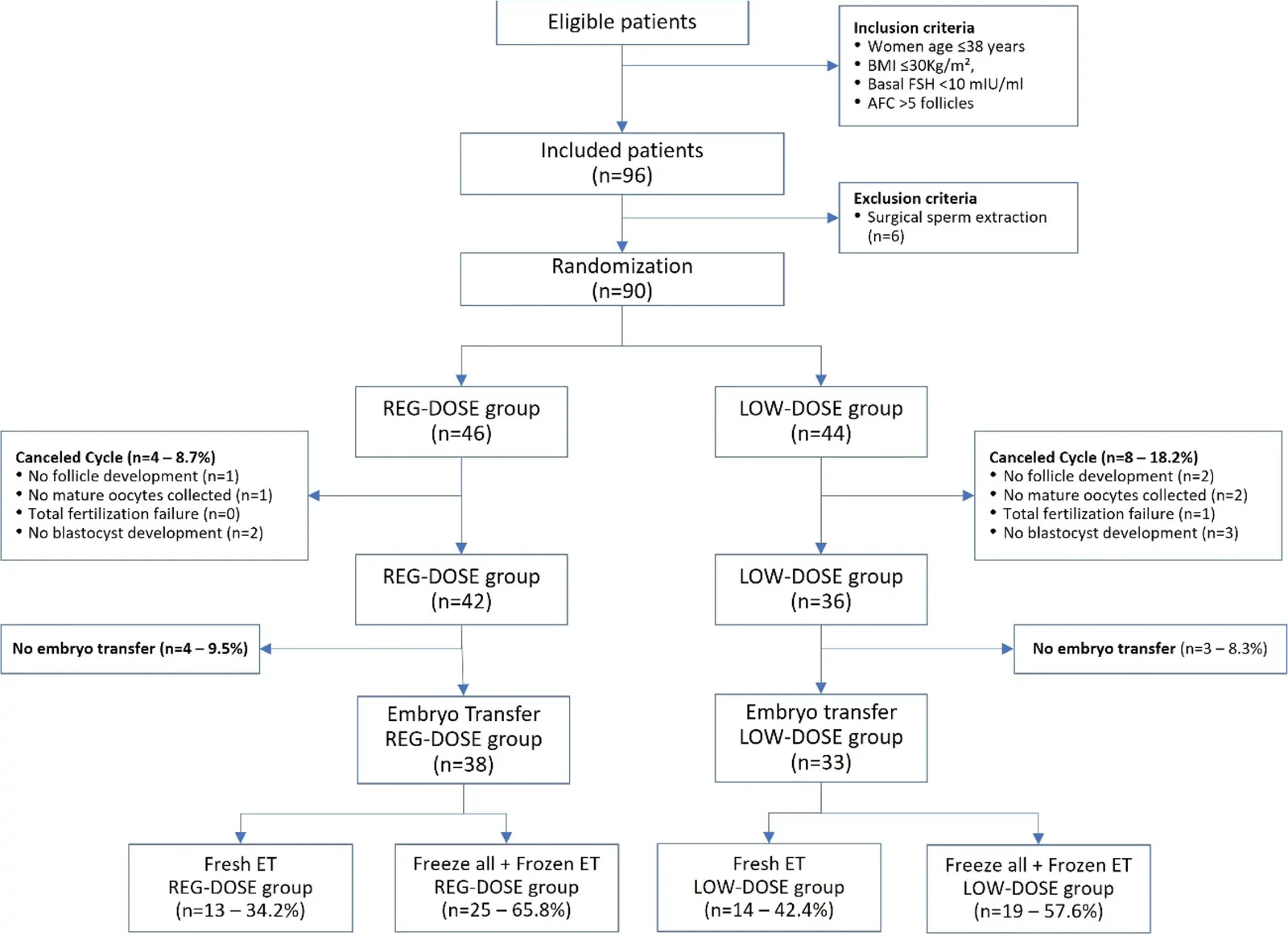
Flowchart of study. BMI, Body Mass Index; AFC, Antral Follicle Count; ET, Embryo Transfer.

### Statistical analysis

The data about demographics and clinical outcomes were annotated. The accumulated clinical pregnancy rate was calculated considering subsequent ET for couples who failed in the first ET and had a second and third ET. Statistical analyses were performed using the JAMOVI 2.3 statistical package. Numeric variables are presented as mean ± Standard Deviation (SD), and categorical variables are presented as frequencies and percentages (%). The Student *t*-test and the Chi-Square test were used as appropriate to detect differences between the two groups. Differences were considered significant at p < 0.05.

## Results

### Demographic and baseline characteristics

The demographic characteristics and baseline clinical data were similar between the REG-DOSE and LOW-DOSE groups, including age, BMI, FSH levels, and antral follicle count. As per protocol, the total gonadotropin dose was significantly lower in the LOW-DOSE group. Once this study included women with good prognosis, the answers to COS were similar demonstrated by the mean number of oocytes and mature oocytes collected (Table 1).

**Table 1:** Demographics and clinical baseline, ovarian stimulation, and embryology laboratory characteristics of the IVF cycles.

	**REG-DOSE mean ± SD or n (%)**	**LOW-DOSE mean ± SD or n (%)**	**p**^a^
**Number of patients**	46	44	
**Demographics and baseline**			
Age (years)	34.83±3.74	34.24±3.28	0.513
Length of infertility (months)	83.61±55.43	78.82±47.59	0.662
BMI (Kg/m^2^)	26.32±3.18	26.46±3.96	0.874
Basal FSH (mIU/ml)	6.11±2.31	6.29±2.15	0.693
Antral Follicle Count (AFC)	12.26±5.28	13.20±5.79	0.421
1st IVF cycle	41/46 (89%)	36/43 (84%)	0.455
**Ovarian stimulation**			
COS duration (days)	9.87±1.82	9.98±1.95	0.787
Estradiol on trigger day (pg/mL)	2866±2148	2351±1636	0.226
Dose of FSH administered	2114±404	1209±383	<0.001
Use of GnRH agonist trigger	12/45 (27%)	13/42 (31%)	0.659
**Laboratory**			
Oocyte pick up	11.02±6.63	8.95±6.56	0.152
Mature oocytes	8.09±5.17	6.88±5.35	0.291
Fertilization rate	76.6%	81.6%	0.337

a Numerical variables were compared using Student’s *t*-test and categorical variables using the Chi-Square test.

### Primary outcome

The absolute number of blastocysts developed (our primary outcome) was very similar, with an average of approximately three blastocysts available for transfer in both groups, confirming the non-inferiority of the LOW-DOSE protocol compared to REG-DOSE (Fig. 3). The REG-DOSE and LOW-DOSE groups were also similar in terms of blastocyst development rate (number of blastocysts formed per fertilized oocyte) (49.9% and 54.8%; p = 0.463) and top-quality blastocyst rate (number of top-quality blastocysts per total number of blastocysts formed (63.4% and 60.1%; p = 0.726).

**Fig. 3:**
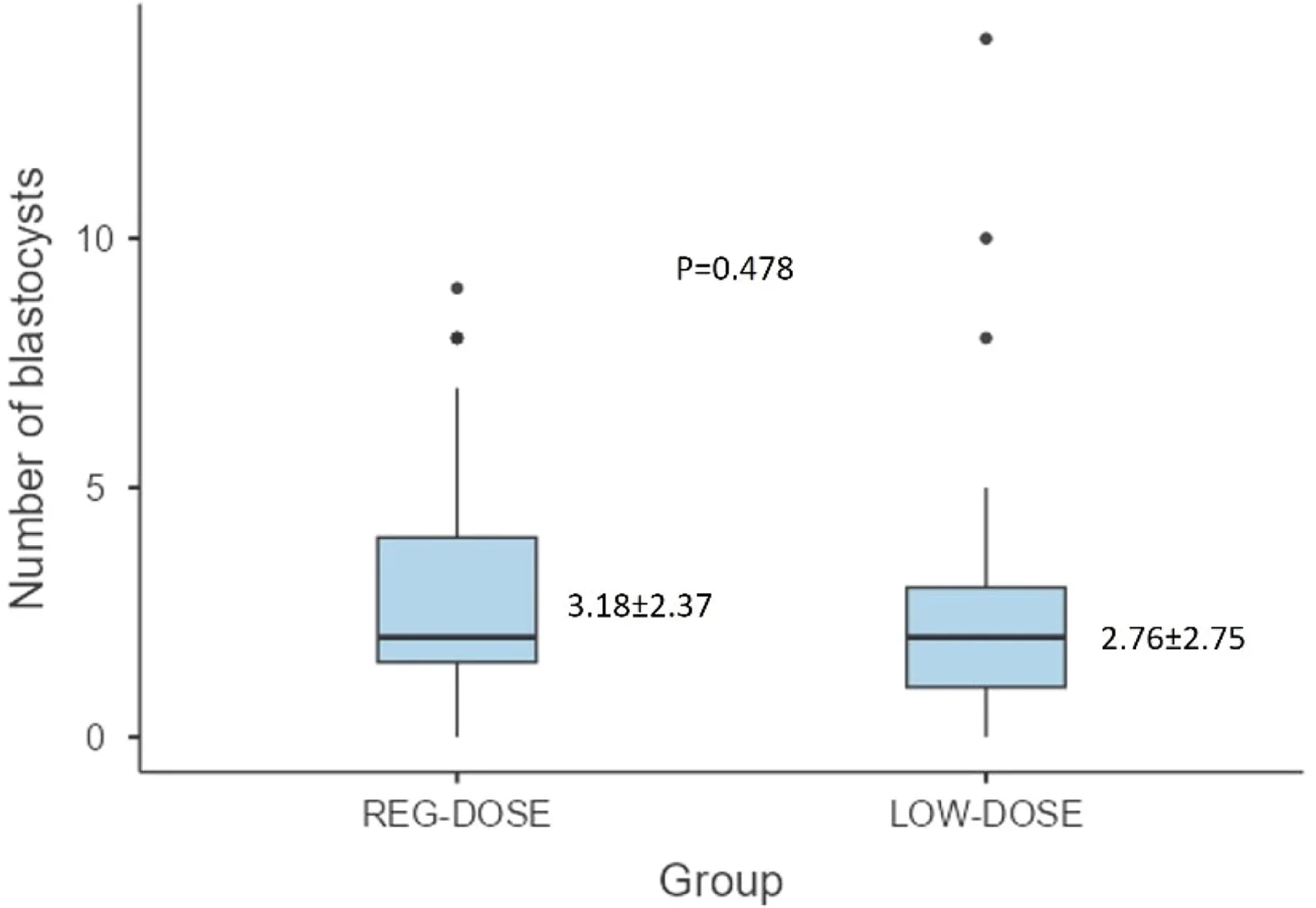
Boxplot graph representing the number of blastocysts developed per group.

### Secondary outcomes

Embryo transfers (ETs) were performed in 38 and 34 patients, among couples in the REG-DOSE (38/46, 82.6%) and LOW-DOSE (34/44, 77.3%) groups respectively. First Embryo Transfers (ETs) were conducted at either the cleavage stage or blastocyst stage in approximately half of the cycles (with 51.5% and 48.5% being blastocyst transfers, respectively; p = 0.477). All subsequent frozen-thawed transfers were carried out at the blastocyst stage. As routine practice, top-quality embryos were always preferentially transferred in the first transfer (76.3% and 79.4%, p = 0.752, respectively), and if available in the subsequent ones. Single Embryo Transfer (SET) was performed in about one-third of first ET for both groups (26.3% and 35.3%; p = 0.409, respectively). Table 2 shows clinical outcomes of the first embryo transfer, with no significant differences between results.

**Table 2:** Clinical outcomes per transfer after the first ET.

	**REG-DOSE Frequency (%)**	**LOW-DOSE Frequency (%)**	**p**^a^
**Number of patients**	38	34	
**Total pregnancy rate**	13/38 (34.2%)	15/34 (44.1%)	0.389
**Clinical pregnancy rate**	11/38 (28.9%)	12/34 (35.3%)	0.564
**Clinical miscarriage**	2/11 (18.0%)	3/12 (25%)	0.692
**Ongoing pregnancy rate**	9/38 (23.7%)	9/34 (26.5%)	0.785
**Multiple pregnancy rate**	1/9 (11.1%)	2/9 (22.2%)	0.527

a Groups were compared by Chi-Square test.

Fifty-four couples failed in the first ET. A second frozen-thawed ET was performed in 12/20 patients (60%) of couples who had surplus embryos available, seven in the REG-DOSE, and five in the LOW-DOSE group. Two patients, one in the REG-DOSE and one in the LOW-DOSE group, underwent a third ET procedure. All frozen-thawed ETs were performed at the blastocyst stage in a hormone-prepared cycle. The cumulative pregnancy rates were 32.4% for REG-DOSE and 30.3% for LOW-DOSE (p = 0.726) (Fig. 4).

**Fig. 4:**
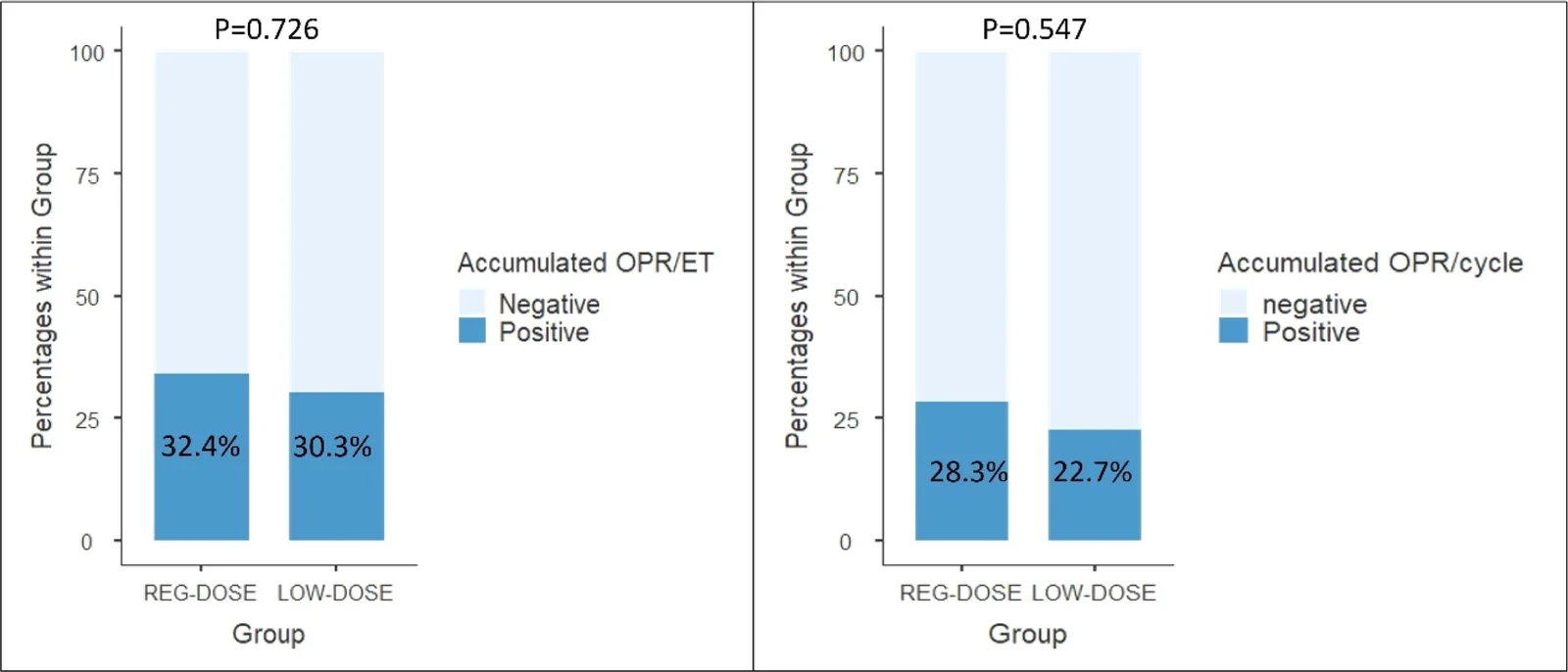
The cumulative ongoing pregnancy outcomes per cycles with embryo transfer (Ongoing pregnancy rate/ET cycle) and ongoing pregnancy outcomes per started cycle (intention to treat analysis).

A few cycles were cancelled in both groups (REG-DOSE: 9% and LOW-DOSE: 16%; p = 0.186). The cycle cancellation motivation was no follicle development, no mature oocytes collected, total fertilization failure, or no embryo development.

### Additional findings

Among the 29 couples in the REG-DOSE group who failed in the first ET, 12 had surplus embryos (41.4%), and among the 24 couples in the LOW-DOSE group who failed in the first ET, 8 had surplus embryos (33.3%; p = 0.547). However, not all couples had the second transfer if they failed the first one. The reasons for not transferring embryos were personal choices, the COVID-19 pandemic or the need for surgery. There were no case reports of OHSS.

## Discussion

Several COS protocols are employed in IVF treatment, with gonadotropin dosages tailored to individual patient profiles.^5^ While high gonadotropin doses has been traditionally used to maximize oocyte retrieval, recent evidence suggests that lower doses can achieve similar clinical outcomes, especially in patients with a good prognosis.^6^, ^7^, ^8^ The results of the present study suggests that a low-dose protocol is non-inferior to regular-dose protocols for COS in good-prognosis patients.

Along with successful clinical outcomes, it is important to highlight other advantages of reduced doses of gonadotropins. The combination of urinary gonadotropins with clomiphene citrate, as used in our study, has an important impact on treatment costs. Furthermore, the low-dose approach provides the additional advantage of lowering overall treatment expenses, thereby improving the accessibility of IVF, particularly within public healthcare systems. This study corroborates the expanding body of evidence advocating for cost-efficient protocols that preserve favorable clinical outcomes while mitigating the economic impact of IVF.

While higher cancellation rates are often seen as a drawback of low-dose protocols,^9^ this study found no significant difference in cancellations between the groups and suggests that this protocol has an opportunity to expand access to IVF. Although individualized protocols using low doses of gonadotropins are not new,^8^ applications of this type of protocol are still rare.^10^ With the growing evidence of the cost-effectiveness of these low-dose protocols, including the results of the present study, it is extremely important to discuss their applicability to the routine of IVF centers, especially government-supported public ones. In addition to the aforementioned benefits, the number of surplus cryopreserved embryos poses both ethical and practical concerns.^11^ Low-dose protocols typically result in fewer remaining embryos, which is advantageous as it reduces storage costs and space requirements while avoiding ethical concerns associated with embryo cryopreserved.

This study was developed during the COVID-19 pandemic, and we couldn't reach the estimated sample size, which represents a limitation. However, the shortfall was less than 10% of the estimated sample size. Not all couples returned for a second ET after a failed first ET, even with cryopreserved embryos available. However, the rate of not returning was similar between groups and likely did not affect the outcome. These couples can still consider future ET.

In summary, this study provides evidence that low-dose gonadotropin protocols for COS in good prognosis IVF patients yield comparable clinical outcomes to regular-dose protocols, with the added benefit of reduced treatment costs. A mean reduction of 900 UI per cycle means approximately R$ 1500,00 of cost reduction. Considering the increasing demand for equitable access to fertility care, especially in resource-limited settings, low-dose protocols represent a viable, cost-conscious alternative without compromising effectiveness. As this was a single-center study, the external validity of our findings may be influenced by center-specific characteristics, including patient selection, ovarian stimulation protocols, and laboratory performance. Although these factors were highly standardized in our center, multicenter studies are needed to confirm the reproducibility and generalizability of these results across different IVF settings.

## Authors’ contributions

All authors contributed to the conception and design of the study. Mayra Satiko Lemos Nakano contributed to the conceptualization, methodology, data curation, and formal analysis. Henrique Penna Fazão contributed to the investigation, data collection, and validation. Tatiana Carvalho de Souza Bonetti contributed to the statistical analysis and interpretation of the results. Edmund Chada Baracat, José Maria Soares Júnior and Pedro Augusto Araújo Monteleone contributed to the supervision and project administration. Mayra Satiko Lemos Nakano drafted the original manuscript, and all authors contributed to reviewing and editing the manuscript. All authors read and approved the final version of the manuscript and agreed to be accountable for all aspects of the work.

## Funding

This research did not receive any specific grant from funding agencies in the public, commercial, or not-for-profit sectors.

## Data availability statement

The datasets generated and/or analyzed during the current study are available from the corresponding author upon reasonable request.

## Declaration of competing interest

The authors have no commercial or financial interests, or any other conflict of interest associated with the publication of this manuscript.
